# Patterns of care and treatment outcomes in older patients with biliary tract cancer

**DOI:** 10.18632/oncotarget.5707

**Published:** 2015-11-09

**Authors:** Anne Horgan, Jennifer Knox, Priya Aneja, Lisa Le, Elizabeth McKeever, Mairead McNamara

**Affiliations:** ^1^ Princess Margaret Cancer Centre, Department of Medical Oncology, Toronto, ON, Canada; ^2^ Present address: University Hospital Waterford, Department of Medical Oncology, Ardkeen, Waterford, Ireland; ^3^ Princess Margaret Cancer Centre, Department of Biostatistics, Toronto, ON, Canada; ^4^ Present address: The Christie NHS Foundation Trust/University of Manchester, Institute of Cancer Sciences, Manchester, UK

**Keywords:** biliary tract cancer, elderly, treatment

## Abstract

**Background:**

Although biliary tract cancers (BTC) are common in older age-groups, treatment approaches and outcomes are understudied in this population.

**Patients and Methods:**

Data from 913 patients diagnosed with BTC from January 1987 to July 2013 and treated at Princess Margaret Cancer Center, Toronto were analyzed. The differences in treatment patterns between older and younger patients were explored and the impact of age, patient and disease characteristics on survival outcomes was assessed.

**Results:**

Three hundred and twenty one patients ≥70 years were identified. Older patients were more likely to receive best supportive care, 40% (*n* = 130), compared to younger patients 26% (*n* = 154); *p* < 0.0001. On multivariable analysis, factors associated with receipt of surgery included stage I/II disease (*p* < 0.0001) and ECOG PS < 2 (*p* < 0.0001). Older age was not associated with lack of surgical intervention. In comparison, older age was associated with non-receipt of palliative chemotherapy (*p* = 0.0007). Similar survival benefit from treatment was seen in older and younger patients. Of 626 patients that underwent either surgery or palliative chemotherapy (*n* = 188), the median survival was 21.1 months (95% CI 19.0–27.9) in patients >70 years of age, and 21.1 months in younger patients (*n* = 438) (95% CI 19.5–24.5).

**Conclusion:**

In this large retrospective analysis, older patients with BTC are less likely to undergo an intervention. However, active therapy when given is associated with similar survival benefits, irrespective of age.

## INTRODUCTION

Tumors of the gastrointestinal tract commonly occur in older people. However, therapeutic strategies for most cancer types have been developed primarily in younger patients, with strict protocol exclusion criteria in clinical trials rendering many older patients ineligible [1]. With the aging of the global population there is growing interest and experience in outcomes for older patients. For the more common cancers, there are elderly-specific randomized controlled trials and robust age-specific subgroup analyses of large studies, the results of which guide our treatment decisions in the clinical setting. However, there is a paucity of such data for rarer tumors. Biliary tract cancers (BTC) incorporating gallbladder cancer, intrahepatic, extrahepatic and perihilar carcinoma, are uncommon, with 10,310 new cases and 3,230 deaths from bile duct cancers (excluding intrahepatic cholangiocarcinoma) and gallbladder cancers in the United States in 2013 [2]. Although the peak age for BTC is the seventh decade [3], treatment approaches and outcomes for BTC are understudied in this population.

We thus undertook this study to compare treatment approaches and outcomes for patients <70 years to those ≥70 years with a diagnosis of biliary tract cancer. Because of the sharp increase in the prevalence of age-related changes between ages 70 and 75 years [4, 5], and with approximately 90% of people showing clinical signs of aging by the age of 70 [4], we chose this as our age cut-off. We sought to identify if older age was associated with non-receipt of active treatment (surgery, chemotherapy) and whether survival outcomes differed between older and younger patients receiving therapy.

## RESULTS

### Overall Population

Nine hundred and thirteen patients were identified, 321 patients aged ≥70 years. Characteristics of the overall population are shown in Table 1. The most common primary was gallbladder (34%) and 90% of patients had an ECOG PS < 2. Nearly half (48%) of patients had advanced disease at diagnosis. In the overall population, 629 (69%) patients received an intervention and 284 (31%) patients received best supportive care alone. Older patients were more likely to receive best supportive care, 40% (*n* = 130), compared to 26% (*n* = 154) in patients <70 years; *p* < 0.0001.

**Table 1:** Baseline characteristics of the overall population

Characteristics	All Patients *n* = 913 Frequency (%)	Best Supportive Care *n* = 284 Frequency (%)	Palliative Chemotherapy *n* = 274 Frequency (%)	Surgery *n* = 355 Frequency (%)
**Female**	452 (50%)	160 (56%)	131 (48%)	161 (45%)
**Age, years**				
median (range)	65.7 (23.7 – 93.7)	68.6 (28.1 – 89.4)	62.0 (23.7 – 93.7)	65.8 (26.6 – 86.4)
<70	592 (65%)	154 (54%)	206 (75%)	232 (65%)
≥70	321 (35%)	130 (46%)	68 (25%)	123 (35%)
**Site**				
Distal Bile Duct	212 (23%)	58 (20%)	53 (19%)	101 (28%)
Gallbladder	310 (34%)	121 (43%)	86 (31%)	103 (29%)
Intrahepatic	200 (22%)	54 (19%)	74 (27%)	72 (20%)
Perihilar	191 (21%)	51 (18%)	61 (22%)	79 (22%)
**ECOG PS**				
<2	795 (90%)	208 (77%)	249 (92%)	338 (97%)
≥2	92 (10%)	61 (23%)	22 (8%)	9 (3%)
Missing	26	15	3	8
**Stage**				
I	137 (15%)	31 (11%)	9 (3%)	97 (27%)
II	178 (20%)	34 (12%)	14 (5%)	130 (37%)
III	159 (17%)	37 (13%)	31 (11%)	91 (26%)
IV	435 (48%)	180 (64%)	219 (80%)	36 (10%)
Missing	4	2	1	1
**CCI**				
0	579 (64%)	158 (57%)	188 (69%)	233 (66%)
1	198 (22%)	72 (26%)	58 (21%)	68 (19%)
≥2	122 (14%)	45 (16%)	27 (10%)	50 (14%)
Missing	14	9	1	4

Abbreviations: ECOG PS: European Cooperative Oncology Group Performance Status; CCI: Charlson Co-morbidity index;

### Surgery

Thirty nine percent (*n* = 232) of younger patients (<70 years) underwent surgery, compared to 38% (*n* = 123) of older patients (≥70 years) (Table 2a). There were no significant differences between the surgical groups in terms of gender, disease site, ECOG PS, or disease stage. However, the older cohort had higher comorbidities (CCI ≥ 2, 20%) compared to younger patients (CCI ≥ 2, 11%, *p* = 0.04). Younger patients undergoing surgery were also more likely to receive adjuvant therapy (chemotherapy/chemoradiation) compared to older patients, 31% vs 20% respectively, though not statistically significant, *p* = 0.08.

**Table 2a:** Characteristics of patients undergoing surgery

Characteristics	Surgery *n* = 355 Frequency (%)	Patients <70 years *n* = 232 Frequency (%)	Patients ≥70 years *n* = 123 Frequency (%)	*p*-value
**Female**	161 (45%)	108 (47%)	53 (43%)	0.58
**Site**				0.21
Distal Bile Duct	101 (28%)	62 (27%)	39 (32%)	
Gallbladder	103 (29%)	62 (27%)	41 (33%)	
Intrahepatic	72 (20%)	51 (22%)	21 (17%)	
Perihilar	79 (22%)	57 (25%)	22 (18%)	
**ECOG PS**				0.28
<2	338 (97%)	223 (98%)	115 (96%)	
≥2	9 (3%)	4 (2%)	5 (4%)	
Missing	8	5	3	
**Stage**				0.82
I	97 (27%)	66 (29%)	31 (25%)	
II	130 (37%)	82 (36%)	48 (39%)	
III	91 (26%)	58 (25%)	33 (27%)	
IV	36 (10%)	25 (11%)	11 (9%)	
Missing	1	1	0	
**CCI**				0.04
0	233 (66%)	160 (70%)	73 (60%)	
1	68 (19%)	44 (19%)	24 (20%)	
≥2	50 (14%)	25 (11%)	25 (20%)	
Missing	4	3	1	
**Adjuvant Chemotherapy**				0.08
Adjuvant Chemotherapy	74 (21%)	57 (25%)	17 (14%)	
Concurrent Chemotherapy / Radiation	21 (6%)	14 (6%)	7 (6%)	
No Adjuvant Chemotherapy	260 (73%)	161(69%)	99 (80%)	

Abbreviations: ECOG PS: European Cooperative Oncology Group Performance Status; CCI: Charlson Co-morbidity index

### Palliative Chemotherapy

Two hundred and eighty four patients with advanced disease received best supportive care (Table 2c), while two hundred and seventy four patients received palliative chemotherapy (Table 2b), 34% of older patients (*n* = 68) and 57% of younger patients (*n* = 206). The most common chemotherapy regimens were gemcitabine and 5FU (46%), gemcitabine/platinum combination (32%) and gemcitabine alone (14%). Older patients undergoing chemotherapy had poorer performance status, ECOG PS ≥ 2, compared to younger patients (16% vs 5%, respectively, *p* = 0.009) and more co-morbidities, CCI ≥ 2, (21% vs 6%, respectively, *p* = 0.003). Older patients were less likely to receive second line therapy compared to younger patients, 16% vs 31%, respectively, *p* = 0.02.

**Table 2b:** Characteristics of patients receiving palliative chemotherapy

Characteristics	Palliative Chemotherapy *n* = 274 Frequency (%)	Patients <70 years *n* = 206 Frequency (%)	Patients ≥70 years *n* = 68 Frequency (%)	*p*-value
**Female**	131 (48%)	101 (49%)	30 (44%)	0.48
**Site**				0.008
Distal Bile Duct	53 (19%)	45 (22%)	8 (12%)	
Gallbladder	86 (31%)	65 (32%)	21 (31%)	
Intrahepatic	74 (27%)	60 (29%)	14 (21%)	
Perihilar	61 (22%)	36 (17%)	25 (37%)	
**ECOG PS**				0.009
<2	259 (92%)	192 (95%)	57 (84%)	
≥2	22 (8%)	11 (5%)	11 (16%)	
Missing	3	3	0	
**Stage**				0.78
I	9 (3%)	7 (3%)	2 (3%)	
II	14 (5%)	9 (4%)	5 (7%)	
III	31 (11%)	24 (12%)	7 (10%)	
IV	219 (80%)	165 (80%)	54 (79%)	
Missing	1	0	1	
**CCI**				0.003
0	188 (69%)	150 (73%)	38 (57%)	
1	58 (21%)	43 (21%)	15 (22%)	
≥2	27 (10%)	13 (6%)	14 (21%)	
Missing	1	0	1	
**First-line palliative chemotherapy**				1.00
Chemotherapy	264 (96%)	198 (96%)	66 (97%)	
Concurrent Chemotherapy/Radiation	10 (4%)	8 (4%)	2 (3%)	
**Second-line palliative chemotherapy**				0.02
Chemotherapy	72 (27%)	62 (31%)	10 (16%)	
Concurrent Chemotherapy/Radiation	4 (1%)	3 (2%)	1 (1%)	
No second-line Chemotherapy	192 (72%)	135 (68%)	57 (84%)	
Missing	6	6	0	

Abbreviations: ECOG PS: European Cooperative Oncology Group Performance Status; CCI: Charlson Co-morbidity index

**Table 2c:** Characteristics of patients undergoing best supportive care

Characteristics	Best Supportive Care *n* = 284 Frequency (%)	Patients <70 years *n* = 154 Frequency (%)	Patients ≥70 years *n* = 130 Frequency (%)	*p*-value
**Female**	160 (56%)	90 (58%)	70 (54%)	0.47
**Site**				0.02
Distal Bile Duct	58 (20%)	29 (19%)	29 (22%)	
Gallbladder	121 (43%)	63 (41%)	58 (45%)	
Intrahepatic	54 (19%)	39 (25%)	15 (12%)	
Perihilar	51 (18%)	23 (15%)	28 (22%)	
**ECOG PS**				0.0007
<2	208 (77%)	124 (86%)	84 (68%)	
≥2	61 (23%)	21 (14%)	40 (32%)	
Missing	15	9	6	
**Stage**				0.005
I	31 (11%)	9 (6%)	22 (17%)	
II	34 (12%)	15 (10%)	19 (15%)	
III	37 (13%)	19 (12%)	18 (14%)	
IV	180 (64%)	110 (72%)	70 (54%)	
Missing	2	1	1	
**CCI**				0.02
0	158 (57%)	98 (65%)	60 (48%)	
1	72 (26%)	33 (22%)	39 (31%)	
≥2	45 (16%)	20 (13%)	25 (20%)	
Missing	9	3	6	

Abbreviations: ECOG PS: European Cooperative Oncology Group Performance Status; CCI: Charlson Co-morbidity index

### Factors associated with receipt of therapy

Factors associated with receipt of surgery on multivariable analysis included stage I/II disease (*p* < 0.0001) and ECOG PS < 2 (*p* < 0.0001) (Table 3). Neither age (*p* = 0.07) nor CCI score (*p* = 0.42) predicted for surgical intervention. In comparison, older age was associated with non-receipt of palliative chemotherapy (*p* = 0.0007), as was female gender (*p* = 0.046), gallbladder primary (*p* = 0.002), stage I/II disease (*p* < 0.0001) and ECOG PS ≥ 2 (*p* = 0.0005).

**Table 3:** Factors associated with interventions in the overall population

	Univariable Analysis				Multivariable Analysis			
	Palliative Chemotherapy vs. Best Supportive Care (OR 95%CI)	*p*-value	Surgery vs. Best Supportive Care (OR (95%CI)	*p*-value	Palliative Chemotherapy vs. Best Supportive Care (OR 95%CI)	*p*-value	Surgery vs. Best Supportive Care (OR 95%CI)	*p*-value
Age, ≥70 years	0.39 (0.27–0.56)	< .0001	0.63 (0.46–0.87)	0.004	0.35 (0.35–0.75)	0.0007	0.70 (0.48–1.04)	0.07
Gender, Female	0.71 (0.51–0.99)	0.04	0.64 (0.47–0.88)	0.006	0.69 (0.48–0.99)	0.046	0.71 (0.49–1.03)	0.07
Site, Gallbladder	0.62 (0.44–0.87)	0.006	0.55 (0.40–0.77)	0.0004	0.55 (0.38–0.81)	0.002	0.79 (0.53–1.17)	0.25
CCI	0.87 (0.76–0.99)	0.03	1.00 (0.91–1.09)	0.95	0.90 (0.78–1.02)	0.10	1.04 (0.94–1.16)	0.42
Stage I / Stage II	0.31 (0.19–0.51)	< .0001	5.97 (4.20–8.49)	< .0001	0.31 (0.18–0.53)	< .0001	6.08 (4.10–9.03)	< .0001
ECOG, ≥2	0.30 (0.18–0.51)	< .0001	0.09 (0.04–0.19)	< .0001	0.37 (0.22–0.65)	0.0005	0.09 (0.04–0.20)	< .0001

Abbreviations: ECOG PS: European Cooperative Oncology Group Performance Status; CCI: Charlson Co-morbidity index

### Overall Survival

**Figure 1:**
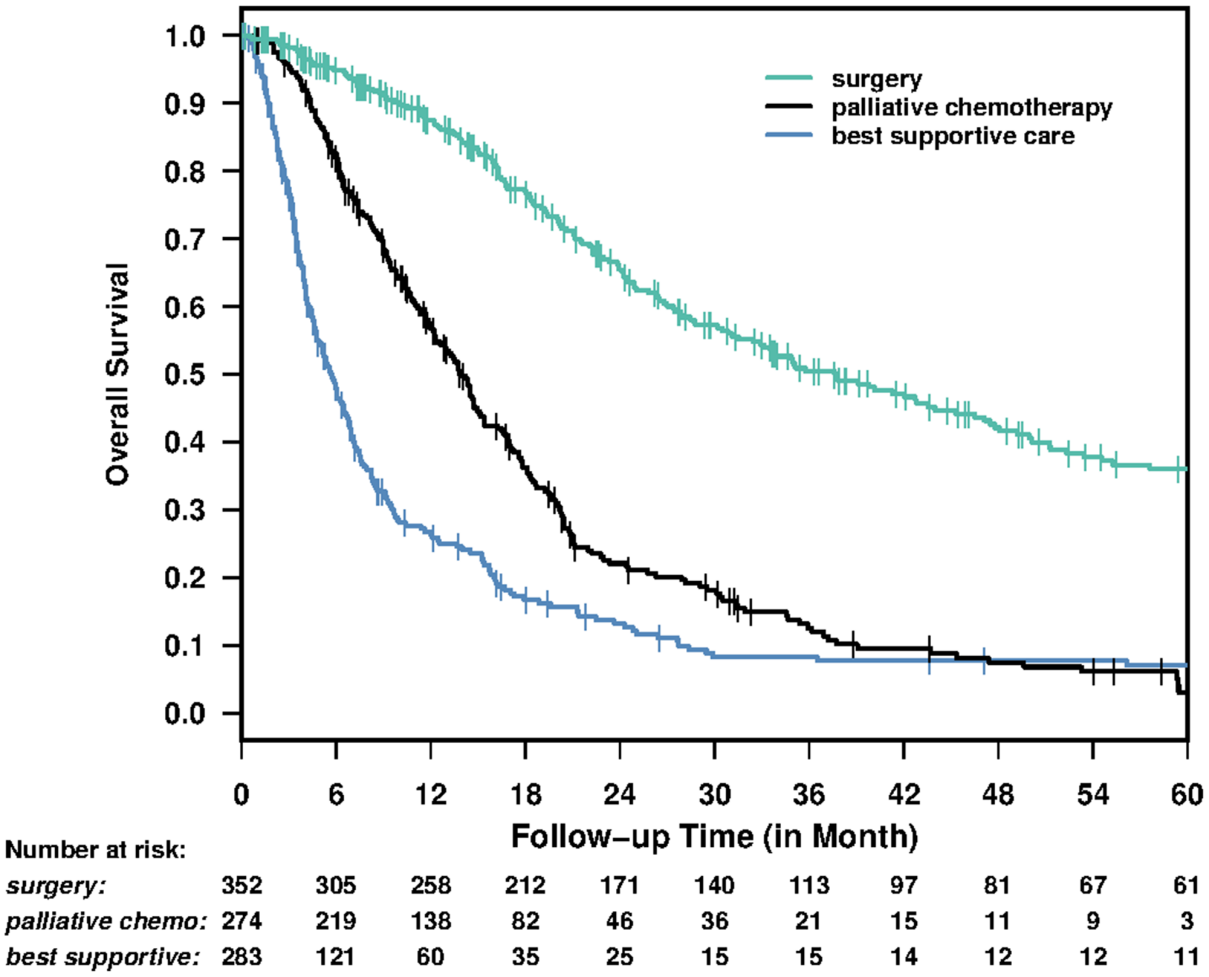
Overall survival for all patients by treatment group

The median follow up time was 12.1 months (range: 0.2–209.0). The median survival time was 37.6 months (95% CI: 31.5–47.1) for surgical intervention, 14.0 months (95% CI: 12.3–15.4) for palliative chemotherapy, and 5.7 months (95%CI: 4.7–6.7) for best supportive care patients, Figure 1.

**Figure 2:**
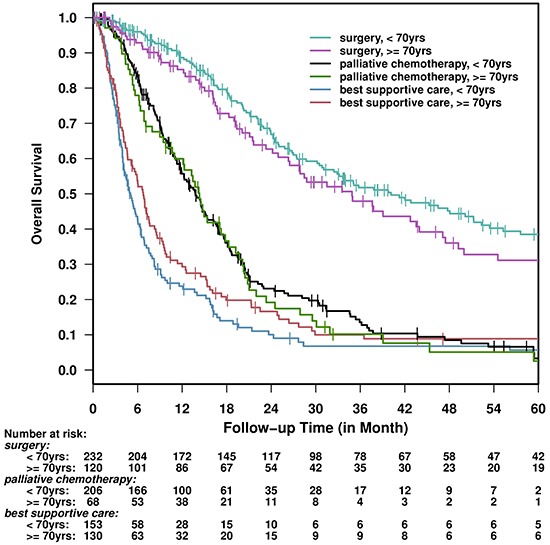
Overall Survival by age and treatment group

Similar survival benefit by treatment was seen in older and younger patients (Figure 2). The median survival for older versus younger patients for BSC was 6.8 (5.2–8.3) versus 5 (4.1–6.2) months; for palliative chemotherapy 14.3 (11–18.3) versus 13.8 months and for surgery 34.9 (26.5–47.1) versus 40.2 (32.5– 52.3) months, respectively.

Table 4 shows the univariable and multivariable survival analyses performed for all patients by age group with stage included in the multivariable analysis as a covariate. The comparison is for stage I/II vs. stage III/IV disease. On multivariable analysis, the hazard ratio associated with surgery vs. best supportive care was 0.29 (95% CI: 0.21–0.41, *p* < 0.0001) in older patients and 0.20 (95% CI: 0.15–0.27, *p* < 0.0001) in the younger; and 0.50 (95% CI 0.36–0.71, *p* < 0.0001) and 0.44 (95% CI: 0.34–0.56, *p* < 0.0001) for palliative chemotherapy (Table 4). Of 626 patients that were selected to receive either surgery or palliative chemotherapy, the median survival was 21.1 months (*n* = 188, 95% CI 19.0–27.9) in patients >70 years of age, similar to younger patients (*n* = 438, median survival 21.1 months, 95% CI 19.5–24.5).

**Table 4:** Univariable and multivariable survival analysis by age group

	Patients <70 years old				Patients ≥70 years old			
	Univariable		Multivariable		Univariable		Multivariable	
	HR (95%CI)	*p*-value	HR (95%CI)	*p*-value	HR (95%CI)	*p*-value	HR (95%CI)	*p*-value
Gender, Female	1.01 (0.89–1.32)	0.41			0.95 (0.74–1.23)	0.72		
Site, Gallbladder	1.37 (1.12–1.68)	0.003	1.10 (0.97–1.48)	0.10	1.03 (0.79–1.34)	0.81		
CCI	0.96 (0.88–1.05)	0.36			0.98 (0.92–1.05)	0.62		
Stage I / Stage II	0.34 (0.29–0.43)	< .0001	0.55 (0.42–0.71)	< .0001	0.40 (0.30–0.53)	< .0001	0.39 (0.29–0.54)	< .0001
ECOG, ≥2	2.90 (2.00–4.22)	< .0001	2.45 (1.67–3.59)	< .0001	2.78 (2.03–3.81)	< .0001	2.05 (1.48–3.84)	< .0001
Treatment								< .0001
Surgery	0.16 (0.13–0.21)	< .0001	0.20 (0.15–0.27)	< .0001	0.27 (0.20–0.37)	< .0001	0.29 (0.21–0.41)	< .0001
Palliative chemotherapy	0.49 (0.39–0.62)	< .0001	0.44 (0.34–0.56)	< .0001	0.72 (0.53–0.99)	0.04	0.50 (0.36–0.71)	< .0001
Best supportive care	−		−		−		−	

Abbreviations: ECOG PS: European Cooperative Oncology Group Performance Status; CCI: Charlson Co-morbidity index

## DISCUSSION

The impact of age on survival outcomes for BTC is uncertain, with a small number of reports suggesting older age negatively affects survival outcomes [6–9] and a number of studies showing no impact [10–17].

In our study, similar proportions of older (38%) and younger (39%) patients underwent surgery. Early stage disease (stage I/II) and ECOG PS < 2 were associated with surgical intervention and survival outcomes did not differ between older and younger patients. In comparison, older age was associated with non-receipt of palliative chemotherapy (*p* = 0.0007). However, similar survival benefit from chemotherapy was observed irrespective of age.

A small number of observational cohort studies have addressed the issue of surgery in older patients with BTC, most of which failed to show age to be an independent risk factor influencing short- and long- term survival after surgery [18]. A retrospective study from Japan reported outcomes for 54 patients ≥75 years and 152 patients <75 years following resection of gallbladder cancer [19]. Approximately 58% of patients in both age groups underwent a radical resection and survival rates were similar between the two age groups. In another small series (*n* = 31) Sawada et al reported outcomes for patients with hilar cholangiocarcinoma undergoing surgical resection [20]. There were no differences in postoperative morbidity rates between younger and older patients, 13% and 33%, respectively (*p* = 0.23) and overall 5-year survival rates were similar. This study confirmed that although surgical resection for hilar cholangiocarcinoma in the elderly is associated with relatively high morbidity rate, it is feasible. A larger study from Korea compared outcomes for older (*n* = 326) and younger (*n* = 205) patients with BTC [21] following treatments including surgery, chemotherapy and radiation therapy. Compared to our population, a lower age cut-off of 65 years was used. There was no difference in the frequency of surgery by age group in this series, and age was not a factor associated with survival in the surgical patients.

There is even less data regarding chemotherapy outcomes in older patients with BTCs. The ABC-02 trial established the combination of gemcitabine and cisplatin as the standard of care for patients with advanced BTC [22]. The BT22 study in Japan was launched at the same time as the ABC-02 study and confirmed a better outcome from combination therapy with cisplatin and gemcitabine versus monotherapy with gemcitabine alone [23]. In the ABC-02 study the median age in the gemcitabine alone group was 63 years and in the combination arm 64 years. In the BT22 study, the median ages were 67 and 65 years, respectively. Although patients up to the age of 85 were included in these studies, subgroup analyses on tolerance and efficacy in the elderly were not performed.

A number of smaller institutional studies assessing outcomes of chemotherapy in older patients have been reported. Kuriyama reported the impact of single agent gemcitabine (*n* = 13) as compared to BSC on survival in 28 patients aged 70 years or over with unresectable BTC [24]. The median overall survival was 9.1 and 2.9 months, for the treated and BSC groups respectively, with a 1 year survival rate of 15% and 7%, respectively. Kou et al compared outcomes for 94 older patients (≥75 years) and 309 younger patients (<75 years) receiving chemotherapy for advanced BTC [25]. There was no difference in the median overall survival between the elderly group (10 months) and younger group (11.5 months) reported.

In the study by Lee et al, older patients (≥65 years) underwent less chemotherapy (*p* < 0.001) than their younger counterparts, however, survival was comparable between the two groups [21].

Limitations of our study include a lack of data regarding surgical procedures performed, although all surgery was done with curative intent. In addition, intrahepatic, extrahepatic and gallbladder cancers were grouped together. However, the numbers of patients with each tumor type were equally distributed by age group and data from the United Kingdom ABC-02 study demonstrated that site of tumour within the biliary tract did not affect survival [26]. Our study also lacks data on toxicity from systemic therapy which is difficult to capture reliably retrospectively. There were only 64 patients ≥80 years and therefore meaningful sub group analysis was not possible in this cohort. Finally, this study includes data from a single-institution. However, Princess Margaret Cancer Center, as part of the University Health Network in Toronto, and the largest cancer center in Canada, is a tertiary referral center with local, regional and provincial referral pathways and thus captures data from a diverse population. Notwithstanding these limitations, this is the largest study (*n* = 913), comparing outcomes in older and younger patients referred to a tertiary referral centre with BTC treated with surgery, chemotherapy and best supportive care and includes patients treated in the era of cisplatin/gemcitabine doublet therapy for advanced disease and thus provides meaningful information which can help guide discussion and decisions regarding treatment in older patients in the clinical setting. Our study confirms that older patients with BTC are less likely to be offered chemotherapy compared to younger patients. However, active therapy results in similar survival benefits, irrespective of age. Given the heterogeneity of the older population, integrating assessments to better biologically stage these patients should lead to better treatment decisions. There is a growing body of evidence supporting the use of comprehensive geriatric assessments in guiding treatment decisions in older patients with cancer [27–32] and in addition, there are assessment tools available to better predict tolerance and toxicity to chemotherapy in this population [33, 34]. Treatment decisions for BTC in older patients should thus not be guided solely by the biological age of the patient, and active management should be considered for this patient population if deemed appropriate following clinical assessment.

## MATERIALS AND METHODS

Data from patients with histologically confirmed biliary tract cancer diagnosed between January 1987 and July 2013 were collected from the Princess Margaret Cancer Centre database on biliary tract cancers. Data collected included baseline characteristics - age, gender, Eastern Cooperative Oncology Group Performance Status (ECOG PS) and co-morbidities reported using the Charlson Co-morbidity index (CCI) [35]. Disease characteristics included site of primary (intrahepatic, hilar, distal bile duct, and gallbladder carcinoma) and disease stage according to the 7^th^ edition of the American Joint Committee (AJCC) on cancer staging system. Patients with carcinoma of the ampulla of Vater were excluded, as these are thought to behave differently to other cancers of the biliary tract [36]. Treatment modalities collected included surgery with curative intent (R0 [negative margins]/R1 [microscopic positive margins]), chemotherapy or best supportive care. Patients receiving active therapy (surgery, chemotherapy) were classified as the intervention group whereas those receiving best supportive care were classified as the non-intervention group. This study was approved by the institutional review board of the Princess Margaret Cancer Centre.

### Statistical analysis

Data were summarized for all patients and by treatment group. Frequency and percentage were reported for categorical variables, and median and range for continuous variables. Among patients who underwent surgery or palliative chemotherapy, Chi-square tests were used to test the differences in demographics and disease characteristics between older (≥70 years) and younger patients (<70 years). A generalized logistic regression was performed to identify factors associated with receiving interventions vs. best supportive care, and variables included in the analysis were age, gender, ECOG PS, CCI score, stage and site of primary. Odds ratio (OR) with 95% confidence intervals (CI) was reported, with an OR > 1 indicating a higher chance of receiving an intervention.

Survival was calculated from the date of diagnosis to date of death or censored at last follow-up date, using the Kaplan-Meier method. Date of death was obtained from patients notes or, when not documented, from the Cancer Care Ontario Registry. The Cox proportional hazards model was used to examine survival outcome by age group. Factors were included in the multivariable analysis if they reached a significance level of *p* < 0.25 on univariable analysis. Hazard ratio (HR) was reported, with a HR < 1 indicating a lower risk of death. Statistical analysis was conducted using SAS v9.3 (Cary, NC).
